# Peer support for smoking cessation: a protocol of systematic review and meta-analysis

**DOI:** 10.1186/s13643-021-01850-y

**Published:** 2021-11-12

**Authors:** Hyun-Ju Seo, Soo Young Kim, Dongah Park, Seung-Soo Sheen, Miyoung Choi, Bo-Hyoung Jang, Su Jung Lee, Youngju Cha

**Affiliations:** 1 College of Nursing, Chungnam National University, Daejeon, Republic of Korea; 2 Department of Family Medicine, College of Medicine, Hallym University, Seoul, Republic of Korea; 3 Kangdong Sacred Heart Hospital, 445 Gil-Dong, Gangdong-Gu, Seoul, 05354 Republic of Korea; 4 National Evidence-based Healthcare Collaborating Agency, Seoul, Republic of Korea; 5 Department of Pulmonology, Ajou University Hospital, Suwon, Republic of Korea; 6 Department of Preventive Medicine, College of Korean Medicine, Kyung Hee University, Seoul, Republic of Korea; 7 College of Nursing, Korea University, Seoul, Republic of Korea; 8 Regulatory Site Service, Covance Korea, Seoul, Republic of Korea

**Keywords:** Peer support, Intervention, Smoking cessation, Systematic review

## Abstract

**Background:**

Peer-support programs are a useful social support strategy for populations trying to quit smoking who are willing to maintain smoking abstinence. This study is a protocol for a systematic review and meta-analysis to assess the effectiveness of peer support for smoking cessation.

**Methods:**

This protocol will be conducted in accordance with the Cochrane Handbook of Systematic Reviews of Interventions 6.2. We will conduct a comprehensive search in the Cochrane Central Register of Controlled Trials, ovidEmbase, PsycINFO, the Cumulative Index to Nursing and Allied Health Literature, ovidMEDLINE, Google Scholar, and Open Grey, as well as the Trials Register of Promoting Health Interventions in EPPI-Centre, ClinicalTrials.gov, the WHO International Clinical Trials Registry Platform, and reference lists of included papers. The review will include randomized controlled trials of peer support interventions aimed to stop smoking in any population. Two reviewers will independently screen and select relevant studies. Version 2 of the Cochrane tool that assesses risk of bias in randomized trials will be used to assess the risk of bias in the included studies. The primary outcomes will be defined as the tobacco abstinence rate and adverse events. If a quantitative synthesis is not appropriate, a synthesis without meta-analysis will be undertaken.

**Discussion:**

This review will provide the best available evidence regarding the effects of peer support interventions to quit smoking. The results from this study will help to inform healthcare providers on the optimal peer support intervention modalities such as intensity, delivery methods, type of support provider, and duration of the intervention.

**Systematic review registration:**

PROSPERO CRD42020196288

**Supplementary Information:**

The online version contains supplementary material available at 10.1186/s13643-021-01850-y.

## Background

Smoking leads to death, illness, and disability and harms nearly every organ of the body [1]. In particular, tobacco kills up to half of its users, and more than 8 million people die as a result of tobacco use each year. Also, about 1.2 million non-smoking people are exposed to second-hand smoke [2]. Smoking continues to be a leading cause of preventable death and disability. Among smoking-related deaths, most were attributable to cancers, cardiovascular disease, and respiratory diseases [3]. In addition, worldwide, the total economic cost of smoking (from health expenditures and productivity losses together) totalled purchasing power parity $1852 billion (USD $1436 billion) in 2012, equivalent in magnitude to 1.8% of the world's annual gross domestic product [4].

Therefore, it is critical to stop smoking as early as possible. However, a large body of evidence suggests that quitting smoking is very difficult. For individuals who try to quit on their own, the average cessation rate is 5% [5]. Furthermore, in a previous national survey in South Korea, more than half the current smokers had attempted to quit smoking; however, most of them failed to quit smoking because of being unable to overcome the urge to smoke in a stressful situation and not possessing the required perseverance to quit smoking altogether [6]. Most smokers tried to quit smoking by themselves, but they failed to stop smoking, therefore, they need a program to support quitting smoking.

Peer support is broadly defined as “a system of giving and receiving help founded on key principles of respect, shared responsibility, and mutual agreement of what is helpful” [7]. Dennis (2003, p. 329) defined peer support within a healthcare context as “the provision of emotional, appraisal and informational assistance by a created social network member who possesses experiential knowledge of a specific behavior or stressor and similar characteristics as the target population” [8].

Although a previous systematic review has already addressed the peer support intervention for socially disadvantaged smokers, such as indigenous peoples, people with severe mental illnesses, and homeless people [9], uncertainty remains about its effectiveness and appropriate intervention modalities for clinically diverse smoking people, including the general population, owing to the complexity of peer support interventions. Therefore, we aim to provide comprehensive evidence of peer support interventions for smoking cessation in tobacco smokers.

## Methods

This review protocol was registered on PROSPERO, the International Prospective Register of systematic reviews, registration number CRD42020196288. The Cochrane Handbook of Systematic Reviews of Interventions (Version 6.2) will guide this systematic review [10]. This study will be reported in accordance with the recommendations in the PRISMA-P guidelines [11].

### Eligibility criteria

#### Participan*ts*

For participants of interest, we will include studies on examining current tobacco smokers among various populations. These will incorporate tobacco smokers, regardless of disease, ethnicity, or socio-economic status, smokers receiving peer support intervention, adolescents, adults, and older adults. We will place no limit on the recruitment setting. Participants will not be required to express an intention to quit smoking at the study intake.

Regarding peer groups of interest, we define peers as people selected to provide support because they had similar or relevant health experiences [12]. Peers can be people who share common characteristics with a specific individual or group, affiliating and empathizing with and supporting each other to promote health and deal with life problems. However, we will exclude families, partners, and parents as peer supporters because they are not classified as general peers.

#### Interventions

Peers are considered to be equal [8] in contrast to traditional healthcare, which distinguishes between providers (trained professionals) and consumers (e.g., families/friends). Peer-support programs are built on collaborative, mutual, and equal partnerships of participants who share their experiences or expertise [13].

We regard peer support intervention as complex intervention; therefore, the elements of peer support intervention are as follows [14]:

1. Use of experiential learning process

This experiential process is a major component of self-help groups, and the evidence provided by self-help research provides further support for beneficial outcomes. Peers in the process of recovery are excellent role models and have much experiential knowledge of dealing with common concerns and problems to offer other peers. Peer providers are particularly adept at negotiating the diversity of systems and agencies on behalf of others, due to their own experiences and encounters with societal and system barriers.

2) Use of mutual benefit

Those who help other peers also gain from this experience as much as they give. This is the primary premise of self-help groups. There is a relatively high level of support for this critical ingredient.

3) Use of natural social support

Natural social support is essentially an inherent element of peer delivered services, much like experiential learning process.

4) Voluntary nature of the service

Choice and self-determination are key philosophies of the consumer movement, which then carry over into the consumer service arena. Individuals who do not want peer service provision will be unlikely to attend these services.

5) Primary control of service by peer service providers

Lotery and Jacobs [15] stated that self-help members’ “retaining control over the functioning, goals and ultimate destiny of the group, is central to the successful functioning of these groups.” Peer-provided services need to be peer driven; otherwise, peers feel disempowered. If peer service providers feel disempowered, their effectiveness is undermined.

Peer support systems can serve as an entry point into the healthcare system for hard-to-reach individuals and, at the very least, serve as a means of providing supportive services for those who would otherwise not engage in treatment.

#### Comparators

Control groups may receive no intervention or standard care, as defined by primary studies.

### Outcomes

#### Primary outcomes

1. Tobacco abstinence rate
  - Short-term abstinence: < 3 months after quitting day [16]
  - Medium-term abstinence: > 3 months and < 12 months after quitting day
  - Long-term abstinence: > 12 months after quitting day [17]

We will use sustained cessation rates in preference to point prevalence, and biochemically validated abstinence in preference to self-report, where available, or where data can be provided by study authors.

2) Adverse events—both adverse events and serious adverse events (number of people experiencing adverse events)

#### Secondary outcomes

1. Change in number of cigarettes smoked per day.

### Study design

We will include various types of randomized controlled trials (RCTs) on peer support interventions for smoking cessation.

In addition, abstracts will be excluded if there is not enough information to assess the risk of bias and/or include in the synthesis.

### Search strategy

We will search the Cochrane Central Register of Controlled Trials, ovidEmbase, PsycINFO, the Cumulative Index to Nursing and Allied Health Literature and ovidMEDLINE. Also, we will search the grey literature in Google Scholar and Open Grey (http://www.opengrey.eu/). The Trials Register of Promoting Health Interventions in EPPI-centre, ClinicalTrials.gov, the WHO International Clinical Trials Registry Platform, and reference lists of included papers will be searched. Medical Subject Headings and text words for peer support and smoking cessation will be used to retrieve related studies. In addition, an RCT search filter will be used to maximize the sensitivity and specificity of searching [18]. Searches will be conducted from inception of each database onwards. The ovidMEDLINE search strategy is provided in Additional file 1.

### Data collection and analysis

#### Selection of studies

We will merge search results using the reference management software Endnote and then transfer results to Covidence (Covidence; Veritas Health Innovation, Melbourne, VIC, Australia) to remove duplicate records and selection. Paired teams of independent review authors will screen the titles and abstracts to identify relevant articles and will subsequently retrieve and examine the full-text articles to assess adherence with the eligibility criteria. If any disagreement for study selection should emerge, a third reviewer (SYK) will be involved to facilitate consensus on study selection. The selection process will be documented in a PRISMA flow diagram [19].

#### Data collection process

Two review authors will pilot the data extraction forms using a small sample of included studies and make appropriate revisions. Two authors will independently extract data using a predetermined and structured data extraction form. Two reviewers will separately perform the data extraction and cross–check the data.

We will extract the following data from each study using a pre-specified form in Covidence: Study design; Population type; Percentage (%) male; Mean age (standard deviation ); Intervention(s) used (if relevant); Comparison used; Outcome measure(s); Length of follow-up; N at baseline and follow-up; Data to calculate risk of quitting tobacco : for each group - N participants in the control group at baseline, N participants in exposure group at baseline, N participants in the exposure group at follow-up, N participants in the control group at follow up; Data to calculate mean difference (MD) in number of cigarettes smoked per day : for each group - mean at baseline and follow up, mean change from baseline to follow-up, and difference in mean change from baseline to follow-up, and variance; Sources of study funding and authors’ declarations of interests. In the case of missing numerical data or key information (including key study descriptive information, primary or secondary outcome data, or risk of bias domains), we will contact study authors for additional information. Where this is not possible, and the missing data pose a serious risk of bias, we will explore the impact of including these studies in the overall assessment of results using a sensitivity analysis.

#### Risk of bias assessment

We will assess the risk of bias for each RCT using Version 2 of the Cochrane tool for assessing risk of bias in randomized trials, composed of the following: Bias arising from the randomization process, Bias due to deviations from intended interventions, Bias due to missing outcome data, Bias in measurement of the outcome, and Bias in selection of the reported result [20].

Two review authors will independently assess the risk of bias for each included study, as outlined in the Cochrane Handbook for Systematic Reviews of Interventions, Chapter 8 [21]. Risk of bias within and across included studies will be illustrated through a risk of bias graph and a risk of bias summary.

#### Data synthesis

The unit of analysis will be the individual participant in parallel-group randomized trials. If cluster-randomized trials are included in the review, we will apply the data properly accounting for the cluster design rather than for a single individual.

#### Quantitative synthesis

For dichotomous data, we will pool risk ratios (RRs) and pool measures of variance calculated for individual studies using a Mantel-Haenszel random effects meta-analysis. For continuous outcome data, we will pool the mean difference or standardized mean difference, with 95% CIs, using a generic inverse variance method [22].

We will use the random-effects model as the interventions and populations are likely to be heterogeneous across included studies. Where raw data are not reported in included studies, but effect estimates and their standard errors are available, we will enter these data directly into Review Manager (RevMan) using the generic inverse variance random-effects model. We will assess the heterogeneity of treatment effects across studies using the *I*^2^ and the Q-statistic [22]. An *I*^2^ value of > 50% will be considered an indication of substantial heterogeneity [23]. Contour enhanced funnel plot will be used to detect any publication bias if more than 10 studies are included in this analysis [17].

#### Non-quantitative synthesis

If meta-analyses are not possible due to substantial heterogeneity, owing to clinical diversity of various types of participants, and modality of interventions, we will consider alternative synthesis methods, including vote counting based on the direction of effect, harvest plots, or an albatross plot [24].

Where statistical test results are available from each study, vote counting might be used, in which the numbers of studies reporting a positive, negative, or null association using a predefined *P* value threshold are counted. Harvest plots have been proposed as an extension of vote counting, providing a graphical tool for displaying the results from each study. In a harvest plot, each study is represented by a bar whose height and appearance convey information related to confidence in the result (e.g., risk of bias), and the bars are grouped by whether the study found a positive, negative, or null association.

When insufficient information is available, we will present results in an albatross plot. The albatross plot is based on minimal statistical information that is usually available from each study, namely, a precise *P* value and a total sample size using Stata 16/SE [25].

#### Subgroup-analyses

If substantial heterogeneity is observed, we will conduct subgroup analyses to investigate the impact of:

1. Population comparison: we will examine whether there is evidence of a difference in effect size between studies in different clinical conditions (e.g., comorbid conditions) and socio-demographic characteristics (e.g., adults, adolescents)
2. Stratified by intervention modality: face-to-face contact (including interventions delivered completely face-to-face or partially face-to-face) versus no face-to-face contact (i.e., via telephone, text messages, virtual reality settings; individual vs. group peer support); intensity of intervention; type of support providers; duration of the intervention

#### Quality of evidence

For each primary outcome, the Grading of Recommendations Assessment, Development and Evaluation guidelines will be used to judge the quality of evidence within the domains risk of bias, publication bias, imprecision, inconsistency, and indirectness [26]. The quality of the evidence can be scored as high quality (very confident that the true effect lies close to that of the estimate of the effect), moderate quality (moderately confident in the effect estimate: the true effect is likely to be close to the estimate of the effect, but there is a possibility that it is substantially different), low quality (confidence in the effect estimate is limited: the true effect may be substantially different from the estimate of the effect), or very low quality (very little confidence in the effect estimate: the true effect is likely to be substantially different from the estimate of effect).

#### Ethical statement

Institutional review board permission is not required for conducting systematic review and meta-analysis.

## Discussion

This review will systematically evaluate the available evidence on the effectiveness of peer support interventions for smoking cessation. By investigating a difference in effect size between studies in diverse smoking populations, intervention modality, intensity of intervention, and duration of the intervention, it is expected that these findings will help inform policy makers, clinicians, or practitioners to plan and implement smoking cessation programs for customized target populations.

This study has several strengths: it may assist practitioners with decision-making strategies to increase smoking cessation and individuals who try to quit tobacco smoking. Furthermore, an improved and more effective approach may be established through this review for interventions in quitting smoking. However, this systematic review also has some limitations: unexplained heterogeneity may arise from the various types of participants and intervention modalities.

## Supplementary Information

**Additional file 1.**

## Data Availability

The datasets used and/or analyzed during the current study are available from the corresponding author on reasonable request.

## References

[1] CDC. Health effects in Smoking & Tobacco Use. https://www.cdc.gov/tobacco/basic_information/health_effects/index.htm Accessed 22 Oct 2021.

[2] World Health Organization. Toabacco. https://www.who.int/news-room/fact-sheets/detail/tobacco Accessed 22 Oct 2021.

[3] Hersi M, Traversy G, Thombs BD, Beck A, Skidmore B, Groulx S (2019). Effectiveness of stop smoking interventions among adults: protocol for an overview of systematic reviews and an updated systematic review. Syst Rev..

[4] Goodchild M, Nargis N, Tursan d'E E (2018). Global economic cost of smoking-attributable diseases. Tob Control..

[5] Livingstone-Banks J, Ordonez-Mena JM, Hartmann-Boyce J (2019). Print based self-help interventions for smoking cessation. Cochrane Database Syst Rev.

[6] Hwang SS. A Fourth-Wave Panel Study for Smokers based on Korea National Health and Nutrition Examination Survey (KNHANES) and its In-depth Analysis. Seoul national university · Korea Centers for Disease Control & Prevention; 2018.

[7] Mead S, Hilton D, Curtis L (2001). Peer support: a theoretical perspective. Psychiatr Rehabil J..

[8] Dennis CL (2003). Peer support within a health care context: a concept analysis. Int J Nurs Stud..

[9] Ford P, Clifford A, Gussy K, Gartner C (2013). A systematic review of peer-support programs for smoking cessation in disadvantaged groups. Int J Environ Res Public Health..

[10] Higgins JPT, Thomas J, Chandler J, Cumpston M, Li T, Page MJ, et al. Cochrane Handbook for Systematic Reviews of Interventions version 6.2. Cochrane; 2021. https://training.cochrane.org/handbook/current Accessed 22 Oct 2021

[11] Moher D, Shamseer L, Clarke M, Ghersi D, Liberati A, Petticrew M, Shekelle P, StewartLA. Preferred Reporting Items for Systematic Review and Meta-Analysis Protocols (PRISMA-P) 2015 statement. Syst Rev. 2015;4(1):1. 10.1186/2046-4053-4-1 PMC4320440 25554246

[12] Dale J, Caramlau IO, Lindenmeyer A, Williams SM. Peer support telephone calls for improving health. Cochrane Database Syst Rev. 2008;(4):CD006903. 10.1002/14651858.CD006903.pub2 PMC7386897 18843736

[13] Repper J, Carter T (2011). A review of the literature on peer support in mental health services. J Ment Health..

[14] Solomon P (2004). Peer support/peer provided services underlying processes, benefits, and critical ingredients. Psychiatr Rehabil J..

[15] Lotery JL, Jacobs MK (1995). The involvement of self-help groups with mental health and medical professionals: the self-helpers’ perspective. Prev Hum Serv..

[16] Karadoğan D, Önal Ö, Şahin DS, Kanbay Y, Alp S, Şahin Ü (2018). Treatment adherence and short-term outcomes of smoking cessation outpatient clinic patients. Tob Induc Dis..

[17] Joo H, Cho MH, Cho Y, Joh HK, Kim JW (2020). Predictors of long-term smoking cessation among smokers enrolled in a university smoking cessation program: a longitudinal study. Medicine (Baltimore)..

[18] Scottish Intercollegiate Guidelines Network. Randomized controll stiral search filter. https://www.sign.ac.uk/what-we-do/methodology/search-filters/ Accessed 22 Oct 2021.

[19] Page MJ, McKenzie JE, Bossuyt PM, Boutron I, Hoffmann TC, Mulrow CD (2021). The PRISMA 2020 statement: an updated guideline for reporting systematic reviews. BMJ.

[20] Sterne JAC, Savović J, Page MJ, Elbers RG, Blencowe NS, Boutron I (2019). RoB 2: a revised tool for assessing risk of bias in randomised trials. BMJ..

[21] Higgins JPT, Eldridge S, Li T (editors). Chapter 23: Including variants on randomized trials. In: Higgins JPT, Thomas J, Chandler J, Cumpston M, Li T, Page MJ, Welch VA (editors). Cochrane Handbook for Systematic Reviews of Interventions version 6.2. Cochrane, 2021. https://training.cochrane.org/handbook/current Accessed 22 Oct 2021.

[22] Deeks JJ, Higgins JPT, Altman DG (editors). Chapter 10: Analysing data and undertaking meta-analyses. In: Higgins JPT, Thomas J, Chandler J, Cumpston M, Li T, Page MJ, Welch VA (editors). Cochrane Handbook for Systematic Reviews of Interventions version 6.2. Cochrane, 2021. https://training.cochrane.org/handbook/current Accessed 22 Oct 2021.

[23] Schroll JB, Moustgaard R, Gotzsche PC (2011). Dealing with substantial heterogeneity in Cochrane reviews. Cross-sectional study. BMC Med Res Methodol..

[24] Campbell M, McKenzie J E, Sowden A, Katikireddi S V, Brennan S E, Ellis S et al. Synthesis without meta-analysis (SWiM) in systematic reviews: reporting guideline. BMJ 2020; 368:l6890. 10.1136/bmj.l6890 PMC7190266 31948937

[25] Harrison S, Jones HE, Martin RM, Lewis SJ, Higgins JPT (2017). The albatross plot: a novel graphical tool for presenting results of diversely reported studies in a systematic review. Res Syn Meth..

[26] Schünemann H, Brozek J, Guyatt G, Oxman A, editor(s). Handbook for grading the quality of evidence and the strength of recommendations using the GRADE approach (updated October 2013). GRADE Working Group, 2013. https://gdt.gradepro.org/app/handbook/handbook.html Accessed 22 Oct 2021.

