# Supplementary material for: Peer support for smoking cessation: a protocol of systematic review and meta-analysis
Source: Syst Rev. 2021 Nov 12;10:296. doi: 10.1186/s13643-021-01850-y (PMC8590240; doi:10.1186/s13643-021-01850-y)
Supplement: Supplementary file 1 — Additional file 1. [file 13643_2021_1850_MOESM1_ESM.docx]

**Appendix 1.** ovid**MEDLINE search strategy** (inclusion dates: From 1948 to 9 June 2020)

|  | **Searches** |
| --- | --- |
| 1 | exp Social Support/ |
| 2 | peer support.mp. |
| 3 | exp Self-Help Groups/ |
| 4 | exp Peer Group/ |
| 5 | peer*.tw. |
| 6 | (lay* adj3 (person* or people* or worker* or person* or advisor* or consultant* or leader* or educator* or tutor* or instructor* or facilitator*)).mp. |
| 7 | (expert* adj3 patient*).mp. |
| 8 | ((support* or career* or ~~caregiver*~~) adj3 (group* or network* or communit*)).mp. |
| 9 | (social* adj3 support*).mp. |
| 10 | (mutual* adj3 (aid* or support*)).mp. |
| 11 | exp Mentors/ |
| 12 | (mentor* or befriend* or buddy or buddies).mp. |
| 13 | 1 or 2 or 3 or 4 or 5 or 6 or 7 or 8 or 9 or 10 or 11 or 12 |
| 14 | exp Smoking Cessation/ |
| 15 | (quit* adj3 smok*).mp. |
| 16 | (smok* adj3 stop*).mp. |
| 17 | 14 or 15 or 16 |
| 18 | 13 and 17 |
| 19 | Randomized Controlled Trials as Topic/ |
| 20 | randomized controlled trial/ |
| 21 | Random Allocation/ |
| 22 | Double Blind Method/ |
| 23 | Single Blind Method/ |
| 24 | clinical trial/ |
| 25 | clinical trial, phase i.pt. |
| 26 | clinical trial, phase ii.pt. |
| 27 | clinical trial, phase iii.pt. |
| 28 | clinical trial, phase iv.pt. |
| 29 | controlled clinical trial.pt. |
| 30 | randomized controlled trial.pt. |
| 31 | multicenter study.pt. |
| 32 | clinical trial.pt. |
| 33 | exp Clinical Trials as topic/ |
| 34 | or/19-33 |
| 35 | (clinical adj trial$).tw. |
| 36 | ((singl$ or doubl$ or treb$ or tripl$) adj (blind$3 or mask$3)).tw. |
| 37 | PLACEBOS/ |
| 38 | placebo$.tw. |
| 39 | randomly allocated.tw. |
| 40 | (allocated adj2 random$).tw. |
| 41 | or/35-40 |
| 42 | 34 or 41 |
| 43 | case report.tw. |
| 44 | letter/ |
| 45 | historical article/ |
| 46 | or/43-45 |
| 47 | 42 not 46 |
| 48 | 18 and 47 |
